# Two decades of global tobacco control: time for a rethink

**DOI:** 10.1007/s11739-025-04131-x

**Published:** 2025-11-05

**Authors:** Karl Fagerstrom, Viktor Mravcik, Andrzej Fal, Guillermo Gervasini, Nicolas Roberto Robles

**Affiliations:** 1 Fagerstrom Consulting, Framnasvagen 8, 18531 Vaxholm, Sweden; 2 Spolecnost Podane Ruce, Brno, Czech Republic; 3 Department of Addictology, First Faculty of Medicine, Charles University, Prague, Czech Republic; 4 Department for Drug Policy Coordination, Office of the Government, Prague, Czech Republic; 5 Polish Society of Public Health, Wrocław, Poland; 6 Collegium Medicum, Warsaw Faculty of Medicine, Cardinal Stefan Wyszynski University, Warsaw, Poland; 7 Department of Medical and Surgical Therapeutics, University of Extremadura, Badajoz, Spain; 8 Institute of Molecular Pathology Biomarkers, University of Extremadura, Badajoz, Spain; 9 Nephrology Department, Hospital Universitario de Badajoz, Badajoz, Spain; 10 Biomedical Sciences Department, Universidad de Extremadura, Badajoz, Spain

**Keywords:** Tobacco, Smoking, Prohibition, Harm reduction, FCTC

## Abstract

Two decades after the adoption of the World Health Organization’s Framework Convention on Tobacco Control (FCTC) and the introduction of its MPOWER implementation package, global declines in smoking prevalence have slowed, casting doubt on the adequacy of current tobacco control strategies. This Points of View article considers the effectiveness of MPOWER and questions the feasibility of nicotine eradication as a public health goal. Drawing on global and regional smoking prevalence data, we argue that the exclusive focus on cessation of nicotine use has reached diminishing returns. In contrast, countries where people who smoke have embraced safer nicotine products such as e-cigarettes, heated tobacco, and oral nicotine have seen accelerated reductions in smoking. We highlight the ethical and practical implications and propose an updated approach to tobacco control that incorporates harm reduction into FCTC governance, enabling clinicians and public health practitioners to provide cessation and risk-reduction advice tailored to individual needs. The issues raised in this article are particularly relevant for internists and general practitioners who encounter the clinical consequences of smoking every day in primary care and internal medicine settings. As the article highlights, without reform, the global target of meaningfully reducing the number of people who smoke will remain out of reach this century. A shift toward pragmatic, science-based strategies is urgently needed to reduce the global burden of smoking-related disease. By framing smoking not only as a public health challenge but also as a pressing clinical concern, the paper underscores the importance of integrating harm reduction perspectives into everyday patient care.

## Introduction

As highlighted in the editorial by Polosa, Rodu, and Farsalinos [1], cigarette smoking remains the leading preventable cause of disease and premature death worldwide. Despite the decades-long pursuit of tobacco control efforts focusing on preventing initiation and promoting cessation, many individuals continue to struggle with long-term abstinence, often cycling through relapse and remission. This persistent burden underscores the need for complementary strategies, including harm reduction, which is formally acknowledged in the World Health Organization’s Framework Convention on Tobacco Control’s Article 1(d) (WHO FCTC).

For internists and general practitioners, the issues raised in this article are particularly relevant as they face the clinical consequences of smoking (ranging from cardiovascular disease to chronic respiratory illness, cancer, and metabolic disorders) every day in primary care and internal medicine settings. We argue that current tobacco control strategies have reached diminishing returns, leaving clinicians on the front lines ill-equipped to manage the persistent burden of smoking-related disease. Understanding the evolving evidence around harm reduction approaches, including the role of safer nicotine alternatives, can equip internists and general physicians with practical tools to better advise patients who struggle with repeated quit attempts or relapse.

This year marks the 20th anniversary of the entry into force of the FCTC, which was developed in response to the globalization of the tobacco epidemic to provide a new legal dimension for international health cooperation. The WHO introduced a set of MPOWER measures in 2007 to help countries implement the provisions of the treaty. The MPOWER package consists of monitoring tobacco use, protecting people from tobacco smoke, offering help to quit smoking, warning about the dangers of tobacco, enforcing bans on tobacco advertising, promotion and sponsorship, and raising taxes on tobacco. According to the 2025 report on the global tobacco epidemic, over 75% of the world’s population (~ 6.1 billion people) is covered by at least one MPOWER measure, but just four countries have achieved a maximum level of MPOWER implementation (Brazil, Turkey, Mauritius, and the Netherlands) [2], while the pace of implementation of MPOWER has slowed [3].

Moreover, successful implementation of MPOWER measures has not translated to corresponding decreases in smoking prevalence or reducing gender gap in tobacco use. The most recent edition of the WHO global report on trends in prevalence of tobacco use shows a slowing pace of declines in tobacco use globally, confirming that the global target of reducing tobacco prevalence by 30% by 2025 (compared to the 2010 baseline) will not be met [4]. In this regard, the WHO’s European Region is the second worst-performing WHO region after the Western Pacific. The WHO projects that tobacco prevalence in Europe will decrease by just 17% between 2010 and 2025, well below the 30% target.

Despite this shortcoming, in 2021 the European Union (EU) adopted a target within its cancer plan to reduce tobacco use to under 5% by 2040. What remains unclear is how the EU plans to meet this ambitious goal. The most recent projections from the Global Burden of Disease (GBD) study show that EU smoking prevalence is estimated to decline from around 25% in 2022 to around 19% by 2050 [5], which is a decrease of ~ 23%. However, to achieve the 5% target, smoking prevalence would have to drop by almost 80%. In other words, the declines in smoking prevalence need to be accelerated by approximately a factor of four. At the current pace, EU’s 2040 target may at best be achieved at some point in the twenty-second century.

The GBD also estimates that the global population of people who smoke will decrease only by around 160 million between 2022 and 2050 (from 1.4 to 1.24 billion, i.e., by 5.7 million per year) [5]. Yet, some have suggested that the phase-out—or eradication—of the commercial tobacco supply is already achievable today [6]. However, we already know that prohibiting the supply of products with resilient demand, such as psychoactive substances, is ineffective in reducing demand and increases health and social harms, especially among vulnerable and marginalized populations [7].

There are few reasons to believe that the calls by some WHO representatives for eradication (i.e., a tobacco-free or nicotine-free world; see, e.g., Dr Rüdiger Krech, WHO Director for Health Promotion, stating during the FCTC’s 10th Conference of the Parties in 2024 that “a well-being society has to be a nicotine- and tobacco-free society,” or WHO awards given to countries that ban less harmful tobacco and nicotine products) are more likely to succeed than an almost 70-year-old attempt to eradicate illicit drug use (i.e., a drug-free world). In this regard, another concerning parallel between the FCTC’s and United Nations’ drug control conventions is becoming apparent: they constitute a highly rigid regulatory system resistant to reform and pose a major obstacle for any country seeking to implement innovative control regimes [8]. Tobacco control should learn from these mistakes, rather than repeat them.

We are far from arguing that current approaches have been ineffective in curbing the tobacco epidemic. They have contributed to reducing smoking prevalence, just not at the pace required to achieve the proposed goals. This raises the question of what additional approaches should be considered by not only policymakers but also clinicians and public health practitioners.

Despite substantial clinical trial evidence showing that nicotine replacement therapy (NRT) is an effective treatment, a recent real-world study in the UK failed to prove that over-the-counter NRT increases quit success [9]. The same study showed that most people who try to stop smoking do so without any support, which is the least effective way to quit. The largest observed effect estimate in this study was for heated tobacco products, while e-cigarettes were the most effective quitting tool at a population level, as they have far greater reach than heated tobacco in the UK. Based on the results, the authors recommend that “quit success rates could be improved by encouraging people to use more effective methods.”

Arguably, stronger implementation of MPOWER measures, especially taxation and cessation support, coupled with greater investments in research and tailored, context-specific interventions, including greater availability of medically approved products for cessation and harm reduction from both pharmaceutical and tobacco industries could improve outcomes.

**Fig. 1:**
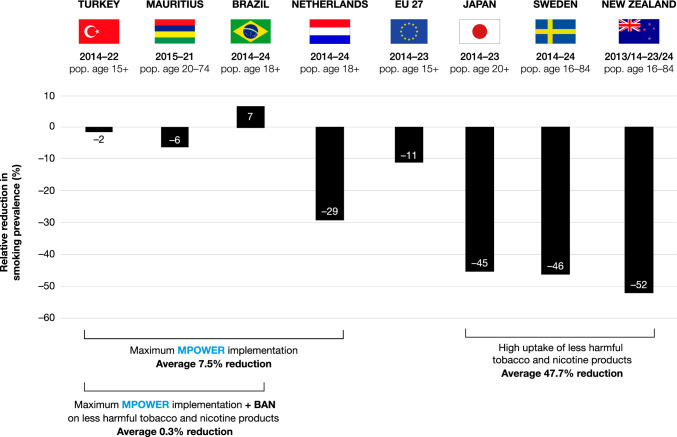
Relative reductions in smoking prevalence over comparable periods in four countries that have implemented MPOWER measures to the highest degree (left), the EU (middle), and three countries with high uptake of less harmful cigarette alternatives such as e-cigarettes (NZ), heated tobacco (Japan), or snus and nicotine pouches (Sweden) (right). Turkey, Mauritius and Brazil completely ban less harmful tobacco and nicotine products. Sources: [10–20]

Moreover, data from countries where less harmful alternatives to cigarettes have been taken up by large numbers of people who smoke show reduction in smoking can be accelerated beyond what can be achieved solely through the implementation of MPOWER measures (Fig. 1). Countries, such as Sweden, New Zealand and Japan have seen smoking prevalence drop by around 50% over the past decade, partially due to smoking being displaced by less harmful forms of nicotine consumption.

In Sweden, where a less harmful cigarette alternative called snus has been available to consumers for decades, the transition from smoked tobacco to a far less hazardous, noncombusted format has been associated with a positive impact on individual and public health [21]. More recently, nicotine pouches, whose toxicological profile is even more favorable than snus and very close to NRT, have appeared on the market in Sweden, the United States and many other countries.

The available body of evidence strongly suggests that clinicians should advise patients that while complete nicotine cessation is ideal, tobacco harm reduction products (e.g., e-cigarettes, heated tobacco products, snus, and nicotine pouches) and long-term NRT use can be valuable tools for people who smoke aiming to reduce or quit combustible tobacco use. They should acknowledge that while the long-term effects of these products are still being studied, the best available evidence shows that they are undoubtedly much safer than traditional cigarettes. Clinicians should support patients in using these alternatives to help them achieve the goal of complete cessation of combustible tobacco product use.

The same body of evidence also offers critical lessons for policymakers. The evidence on MPOWER’s impact on smoking prevalence is not encouraging. At the current rate, smoking prevalence will not meet the WHO’s 2030 objective or individual country goals of reducing smoking prevalence below 5%. There will likely be a demand for nicotine, rather than any particular form of taking it, that persists well into the future. This means that strategies that rely on nicotine cessation are unlikely to yield rapid success. However, smoking cessation strategies that do not rely on achieving nicotine abstinence are far more likely to work rapidly and at scale. Therefore, policymakers should consider reducing smoking—the most dangerous form of tobacco consumption—as the most important measure of policy success. The WHO and FCTC should reconsider their dogmatic and unscientific policy recommendation of banning safer cigarette alternatives (while deadly cigarettes remain legal everywhere) or recommending that governments regulate or tax these products as if they were as dangerous as cigarettes. Such policy harms nicotine users, promotes misinformation, and protects the cigarette industry. It is deeply unethical. Instead, the WHO and FCTC should work alongside countries that have appropriate experience in regulating these products to develop a set of guidelines that can be followed by FCTC members that lack adequate capacity.

The current policy gaps within the FCTC framework and MPOWER implementation have direct consequences for clinical care and population health. For clinicians, the absence of supportive regulatory environments for safer nicotine products limits their ability to offer practical harm reduction advice tailored to individual needs, particularly for patients who are unable or unwilling to quit nicotine entirely. Public health practitioners face the challenge of designing effective programs within restrictive policy frameworks that fail to account for real-world patterns of nicotine use. For policymakers, a singular focus on nicotine cessation has created blind spots in strategy, delaying the potential gains of alternative interventions. As a result, smoking prevalence declines are stalling, and health systems continue to bear the burden of smoking-related disease and expenditure. Without urgent policy reform that acknowledges the nicotine product risk continuum, clinicians and public health actors are left with outdated tools, while populations continue to suffer preventable harm.

A pragmatic path forward should embed harm reduction principles into the next decade of FCTC evolution, as shown in Table 1.

**Table 1:** Proposed Steps for Embedding Harm Reduction into FCTC Evolution

Step	Action	Purpose / Expected Impact
1. Reset scientific insights	Convene WHO-led (e.g., by WHO Chief Scientist) listening sessions and commission fresh scientific advice with broad international representation	Ensure evidence-based, independent review of harm reduction science
2. Improve reporting	Encourage FCTC members to improve tracking of smoking prevalence, uptake of lower-risk products, and monitor diversion from smoking	Generate comparable global data on progress in reducing smoking
3. Create a parallel track	FCTC Conference of the Parties (COP) to establish a formal harm reduction track, distinct from combustible tobacco regulation	Allow regulatory flexibility for reduced-harm nicotine products
4. Develop policy guidelines	Draft guidelines covering product quality, risk communication, taxation, and regulation	Promote consistency, safety, and responsible governance of reduced-harm products
5. Develop clinical guidelines	Draft guidelines and manuals for clinical management and counselling on smoking cessation using reduced-risk nicotine products, adaptation of screening and diagnostic tools to reduced-risk nicotine products. Consider integration into training curricula for clinicians	Equip clinicians with evidence-based tools that support both complete cessation and harm-reduction pathways, reduce misinformation, and improve patient outcomes by incorporating reduced-risk products into treatment strategies

These steps could allow FCTC signatories to modernize their tobacco control strategies without abandoning core treaty goals, ultimately accelerating the decline in smoking-related disease and death.

The first 20 years of the FCTC implementation have not worked as intended, with smoking prevalence declines slowing rather than accelerating. Clearly, the global tobacco control policy needs to be adjusted over the next 20 years to ensure that the dire projection of 1.2 billion people still smoking in 2050 does not come to pass. Evidence suggests that complete cessation of all forms of nicotine use will not be achieved in the foreseeable future. However, substantially reducing the number of people who use the most hazardous form of nicotine delivery—smoking —is possible if more people who smoke or would smoke can be diverted to less hazardous forms of nicotine use.

## Research involving Human Participants and/or Animals

The authors declare that the work reported herein did not require ethics approval because it did not involve animal or human participation.
